# The Plant Nuclear Envelope and Its Role in Gene Transcription

**DOI:** 10.3389/fpls.2021.674209

**Published:** 2021-04-30

**Authors:** Jade Bishop, Hetty Swan, Francesco Valente, Hans-Wilhelm Nützmann

**Affiliations:** The Milner Centre for Evolution, Department of Biology and Biochemistry, University of Bath, Bath, United Kingdom

**Keywords:** nuclear envelope, plants, transcription, epigenetics, chromosome organisation

## Abstract

Chromosomes are dynamic entities in the eukaryotic nucleus. During cell development and in response to biotic and abiotic change, individual sections as well as entire chromosomes re-organise and reposition within the nuclear space. A focal point for these processes is the nuclear envelope (NE) providing both barrier and anchor for chromosomal movement. In plants, positioning of chromosome regions and individual genes at the nuclear envelope has been shown to be associated with distinct transcriptional patterns. Here, we will review recent findings on the interplay between transcriptional activity and gene positioning at the nuclear periphery (NP). We will discuss potential mechanisms of transcriptional regulation at the nuclear envelope and outline future perspectives in this research area.

## Introduction

The eukaryotic nucleus is defined by the presence of the nuclear envelope (NE) acts as a physical barrier dividing the nuclear content from the cytoplasm (Hetzer, 2010). The NE creates an intranuclear environment that shields the genome, enabling coordinated processes, including DNA duplication and transcription (Lammerding and Wolf, 2016). Within the nucleus, the spatial organisation of eukaryotic genomes and association with the NE has been firmly associated with transcriptional regulation (Fedorova and Zink, 2008; van Steensel and Belmont, 2017).

Here, we will discuss the nuclear envelope in plants and its association with different patterns of gene expression. We will compare structure and function of the plant NE with its counterparts in other eukaryotes. We will focus on our current understanding of the link between gene positioning at the NE and transcriptional activity and outline future directions in the field.

## Organisational Principles and Functions of the Nuclear Envelope

The NE is organised into two lipid bilayers, the outer nuclear membrane (ONM) and the inner nuclear membrane (INM), divided by a luminal perinuclear space and the nuclear pore complex (NPC; Goodchild et al., 2015; Meier et al., 2017). The ONM is classified as an extension of the endoplasmic reticulum (ER), and ribosomes are attached to its cytoplasmic surface. Protein content of the ONM is largely shared with the ER (Foresti et al., 2014; Meier et al., 2017). The INM encloses the nucleoplasm and shows a more distinct protein composition (Foresti et al., 2014; Meier et al., 2017). The ONM and INM are connected at the NPCs. These protein channels consist of multiple proteins called nucleoporins (Tamura et al., 2010; Hoelz et al., 2011).

The main role of the INM and ONM is to ensure a spatial continuum between the NE, the cytoplasm, and the nucleoplasm (De Magistris and Antonin, 2018). NPCs act as a bidirectional transport system for macromolecules, which relocate between the nucleus and cytoplasm (Tamura et al., 2015; Meier et al., 2017; De Magistris and Antonin, 2018). Furthermore, NPCs have been implicated in chromatin organisation, transcriptional and post-transcriptional regulation of gene expression, and the progression of the cell cycle (Lince-Faria et al., 2009; Tan-Wong et al., 2009; Ahmed et al., 2010; Kylberg et al., 2010; Strambio-De-Castillia et al., 2010; Vaquerizas et al., 2010; Smith et al., 2015; Tamura et al., 2017). The perinuclear space serves as ion storage and linkage site for interacting proteins from ONM and INM. As such, it functions in nuclear signalling and force transmission between cytoplasm and nucleoplasm (Razafsky and Hodzic, 2009; Starr, 2009; Capoen et al., 2011; Mauger, 2012). It is the site of interaction between linker of nucleoskeleton and cytoskeleton (LINC) complexes that directly link components of the cytoplasm and nucleoplasm (Rothballer and Kutay, 2013).

In metazoan, a dense layer of protein filaments called the nuclear lamina structurally supports the NE underneath the INM (de Leeuw et al., 2018; Alvarado-Kristensson and Rossello, 2019; Tenga and Medalia, 2020). Nuclear lamina composition varies across the eukaryotes. For example, lamin proteins which represent the major constituents of the animal lamina have not been identified in plants (Meier et al., 2017). Instead, three types of plant-specific proteins, CROWDED NUCLEI (CRWN) or nuclear matrix constituent protein (NMCP), KAKU4 and nuclear envelope-associated proteins (NEAPs) have been suggested to function as lamin-like proteins in plants (Masuda et al., 1997; Ciska and de la Espina, 2014; Goto et al., 2014; Pawar et al., 2016; McKenna et al., 2021). Beyond its scaffolding role, the nuclear lamina is of functional importance in chromatin folding, DNA repair, and gene transcription (Burke and Stewart, 2013).

All elements of the NE exhibit high structural variability and may re-organise during cellular differentiation and division. Loss of function of NE components may cause severe diseases in humans and may lead to developmental and signalling defects in plants (Hatch and Hetzer, 2014; Meier et al., 2017; Tang et al., 2020).

## Chromatin Organisation at the NE

Components of the NE control nuclear shape and are directly linked to the nucleoplasmic chromatin. As such, it has a dominant role in defining the organisation of chromosomes in the nuclear space and takes in an important position in the transcriptional regulation of gene expression.

Typically, transcriptionally inactive heterochromatic regions of the genome are located adjacent to the NE. In animals, such ‘lamina associated domains’ (LADs) possess distinct properties and are specifically localised towards the NE. LADs, usually 10 Kb–10 Mb in size, comprise a significant part of the genome and incorporate around a third of the human and mice genomes. LADs are rich in repressive histone marks and gene sparse (Guelen et al., 2008; Kind et al., 2015; van Steensel and Belmont, 2017). LADs may associate stably as well as transiently with the nuclear lamina. Constitutive LADs (cLADs) interact with the nuclear lamina across cell types while facultative LADs (fLADs) are localised at the nuclear lamina in distinct cell types (Peric-Hupkes et al., 2010; Meuleman et al., 2013). cLADs are enriched in repetitive elements and depleted in gene content compared to fLADs (Meuleman et al., 2013).

Despite the absence of nuclear lamin proteins in plants, recent studies have shown that the genome of *Arabidopsis thaliana* is organised non-randomly at the nuclear periphery (NP), in a similar manner to that of LADs in animals. Regions which are associated with the NP in plants have been termed plantLADs (pLADS; Bi et al., 2017; Hu et al., 2019). Heterochromatic domains of centromeric and pericentromeric regions, also called chromocenters, as well as 10–20% of chromosomal arms have been reported to associate with the nuclear periphery in *A. thaliana* (Fransz et al., 2002; Bi et al., 2017; Hu et al., 2019). Like LADs, pLADs are characterised by an enrichment for heterochromatin and transposable elements. However, unlike LADs, pLADs identified in chromosomal arms of *A. thaliana* are neither gene poor nor A/T rich (Bi et al., 2017; Hu et al., 2019). pLAD composition remains largely stable across different plant organs (Bi et al., 2017).

In animals, chromatin is anchored at the NE by lamins and INM associated proteins. B-type lamins, for example, interact with lamin B receptors. These receptors bind to the heterochromatic histone marks H3K9me3 and H4K20me2 *via* their heterochromatin binding and tudor domains (Hirano et al., 2012; Gruenbaum and Foisner, 2015). A-type lamins interact with proteins of the INM and the nucleoplasm. Furthermore, A-type lamins have been shown to associate with GAGA DNA sequence motifs along chromosomes (Zullo et al., 2012; Gruenbaum and Foisner, 2015). Lamins themselves can directly bind to DNA and histones (Taniura et al., 1995; Stierle et al., 2003; Mattout et al., 2007).

In plants, CRWN proteins have been suggested as mediators of pLAD recruitment to the NE (Hu et al., 2019; Sakamoto, 2020). *crwn* mutants display large scale changes in chromatin organisation. In *crwn* mutants, chromatin positioning at the nuclear periphery is significantly altered (Hu et al., 2019). Additionally, *crwn* mutants show an increased chromosomal compaction and disruption of chromocenter organisation (Wang et al., 2013; Grob et al., 2014). By chromatin immunoprecipitation, an interaction of CRWN1 proteins with chromatin has been shown (Hu et al., 2019). Furthermore, CRWN1 proteins have been shown to bind the transcription factor (TF) NTL9, a transcription factor involved in the regulation of plant immunity (Guo et al., 2017). Another candidate for chromosome anchoring at the plant NE is the transcription factor bZIP18, which has been suggested to interact with NEAP1 proteins (Pawar et al., 2016).

## Transcriptional Pattern at the NE

The association of chromatin to the NE has profound implications on transcriptional activity of genes. LADs are largely transcriptionally inactive (Guelen et al., 2008). Introduction of reporter genes into LADs is accompanied by reduced transcriptional activity compared to reporter activity in non-LAD locations (Akhtar et al., 2013). Artificial targeting of genes towards the NE has been shown to cause transcriptional downregulation (Finlan et al., 2008; Reddy et al., 2008). In accordance, release from the nuclear lamina may lead to de-repression of genes (Shevelyov et al., 2009; Kohwi et al., 2013; Chen et al., 2014). Like LADs, pLADs are also characterised by low transcriptional activity (Bi et al., 2017).

Peripheral location of genes at the NE, however, may also lead to increased gene expression. For example, the NPC has been described as a site of high transcriptional activity (Strambio-De-Castillia et al., 2010; Vaquerizas et al., 2010). It is suggested that this trait is based on an enhanced efficiency of mRNA export from the nucleoplasm to the cytoplasm. Nucleoporins of the NPC have been implicated in the recruitment of chromosomal regions to the NPC across eukaryotes. In yeasts, binding of nucleoporins to chromatin modifying complexes with positive function in transcriptional activation is shown to be involved in the upregulation of the NE associated *GAL* gene cluster (Cabal et al., 2006). In *A. thaliana*, fusion of the LacI DNA binding protein to the nucleoporin Seh1 resulted in peripheral localisation of the LacO-Luciferase reporter construct and increased reporter activity (Smith et al., 2015).

**Figure 1:**
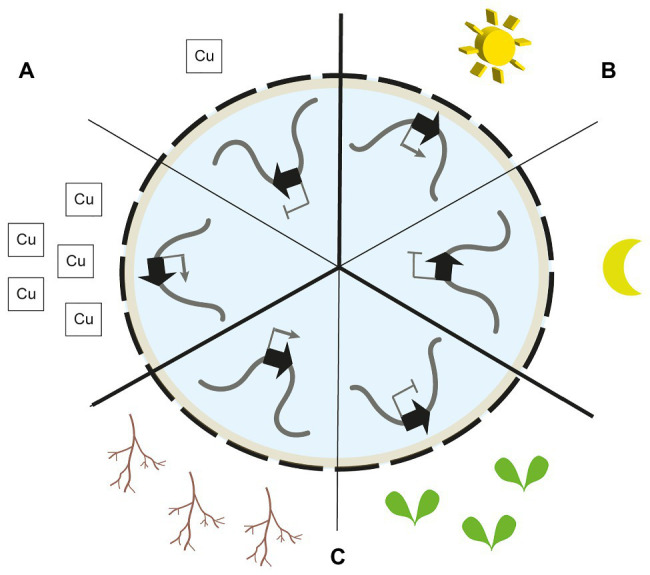
Nuclear gene re-positioning in response to environmental and developmental signals. **(A)** The copper-associated (*CA*) gene cluster is located at the nuclear envelope (NE) upon plant exposure to copper. Peripheral localisation is accompanied by transcriptional activation. In the absence of copper, the cluster is located away from the NE and silenced (Sakamoto et al., 2020). **(B)** The light-induced *CAB* gene cluster is located at the NE upon plant exposure to light. In absence of light, the cluster is located away from the NE and silenced (Feng et al., 2014). **(C)** The silenced thalianol biosynthetic gene cluster is located at the nuclear periphery (NP) in leaf cells. In roots, when transcriptionally active, the cluster is positioned away from the nuclear periphery (Nützmann et al., 2020).

Furthermore, up to 10% of genes encoded in LADs show high expression levels (Guelen et al., 2008; Peric-Hupkes et al., 2010; Wu and Yao, 2017). It is suggested that these genes are driven by promoters that are more resistant to the repressive chromatin environment at the NE and bound by pioneering transcription factors. They may also be preferentially localised at LAD sites with weak NE association (Leemans et al., 2019). Increased transcriptional activity has also been linked to NE association *via* binding to CRWN proteins. A cluster of coordinately expressed copper-associated (*CA*) genes is located towards the nuclear periphery upon transcriptional induction (Figure 1). Chromatin integration labelling (ChIL) confirmed association of the *CA* genes to CRWN1 proteins. Indeed, loss of CRWN1 and CRWN4 results in lower transcription levels of *CA* genes and reduced NE association (Sakamoto et al., 2020).

Such transient re-localisation towards the NE has also been observed for light-responsive genes in *A. thaliana* (Figure 1). The *CAB* gene cluster encoding chlorophyll a/b-binding proteins as well as the *RBCS1A*, *GUN5*, and *PC* genes are re-positioned from the interior of the nucleus towards the periphery upon light exposure to seedlings (Feng et al., 2014). The peripheral localisation of these genes is accompanied by an increased transcriptional activity. Interestingly, the *CAB* gene cluster is re-localised towards the nuclear periphery before the highest transcription levels are reached, thus, showing that chromatin repositioning precedes full transcriptional activation. In photoreceptor mutants, the dynamic re-positioning of the *CAB* gene cluster is lost (Feng et al., 2014).

Biosynthetic gene clusters in *A. thaliana* show the opposite direction in nuclear re-positioning associated with their transcriptional activity (Nützmann et al., 2020; Figure 1). Silenced biosynthetic gene clusters are located at the nuclear periphery while active clusters are located away from the periphery. Such clusters encode biosynthesis genes of specialised metabolic pathways and are exclusively transcribed in root tissues and silenced in the aerial organs of the plant. Chromosome conformation capture and 3D DNA FISH analyses showed that silenced biosynthetic gene clusters are associated with the chromocentric sections of chromosomes in close proximity to the NE. Interestingly, silenced clusters are delineated by H3K27me3 histone marks, and loss of PRC2 activity leads to reduced chromocentric localisation of the loci (Yu et al., 2016; Nützmann et al., 2020).

## Discussion

The link between NE association of genes and transcriptional activity is firmly established. However, how positioning of genes at the NE leads to distinct transcriptional outputs in plant nuclei remains largely unknown (Parry, 2015; Santos et al., 2020).

**Figure 2:**
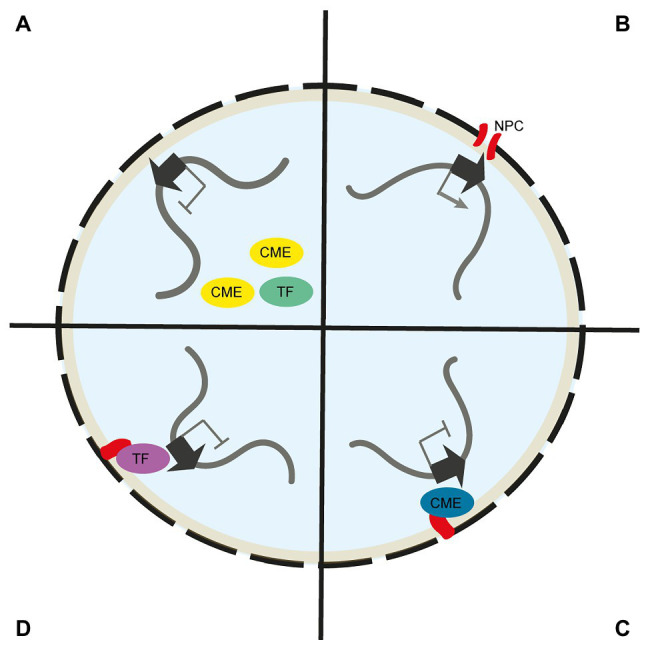
Mechanisms of gene regulation at the nuclear envelope. **(A)** Chromatin modifying enzymes (CMEs) and transcription factors (TFs) with gene activating function are excluded from the NE. As such, peripheral chromosome regions are devoid of active chromatin marks and activating TFs. **(B)** Chromosome regions are recruited to the nuclear pore complex (NPC) *via* nucleoporin interaction. At the NPC, mRNA export is streamlined and loci may interact with NPC associated CMEs. **(C)** Chromosome regions at the NE are exposed to CMEs with gene silencing activity, such as H3K9 methyltransferases, that are directly linked to the NE. **(D)** Chromosome regions at the NE are exposed to repressive TFs that are directly linked to the NE.

In animals, specific binding and increased local concentrations of chromatin modifying enzymes (CME) at the NE have been suggested as key mechanistic factors in gene silencing at the nuclear periphery (Harr et al., 2016; van Steensel and Belmont, 2017). The animal INM protein emerin, for example, directly interacts with the histone deacetylase HDAC3 and modulates its activity (Demmerle et al., 2012). As such, a transient contact of a gene with the NE may result in changes to its histone modifications. Higher abundance of histone lysine 9 methyltransferases at the nuclear periphery may promote heterochromatin formation of NE associated chromosomal segments (Towbin et al., 2012; Gonzalez-Sandoval et al., 2015). Similarly, chromatin modifiers that act positively on transcription, e.g., histone acetylation complexes, are suggested to be actively retained in euchromatic areas of the nucleus and are as such excluded from the NE (Cabianca et al., 2019). Localisation within heterochromatin at the nuclear periphery may also prevent access of activating transcription factors to its target loci or recruitment of genes to transcription factories in the interior of the nucleus (Yao et al., 2011; van Steensel and Belmont, 2017; Figure 2). Furthermore, it has been suggested that gene positioning within LADs may prevent access of gene promoters to enhancers located outside of LADs (Yaffe and Tanay, 2011; Amendola and van Steensel, 2014). Active retention of transcription factors at the nuclear periphery and away from target chromatin may represent another mechanism of NE associated gene regulation (Mirza et al., 2019).

In plants, pioneering work by Mikulski et al. (2019) showed that CRWN1 associated with the PROLINE-TRYPTOPHANE-TRYPTOPHANE-PROLINE (PWWP) INTERACTOR OF POLYCOMBS1 (PWO1) protein, a component of the Polycomb Repressive Complex 2 (PRC2). As such, it is suggested that the localisation of PWO1 links PRC2 to the NE and thus promotes the formation of facultative H3K27me3-marked chromatin at the NE (Mikulski et al., 2019). Indeed, chromosome simulations support the important role of H3K27me3 in chromosome organisation in *A. thaliana* (Di Stefano et al., 2021). Furthermore, different DNA methylation pathways are suggested to target transposable elements at the periphery and interior of the *A. thaliana* nucleus (Bi et al., 2017). This may indicate a mechanistic role for distinct DNA methylation pathways in the silencing of transposable elements at the NE in plants.

A largely unexplored aspect of NE-related influence on gene expression is how mechanical stress put on the exterior of the cell and mediated by the NE affects transcription. LINC complexes link the cytoskeleton with the nucleoskeleton (Rothballer and Kutay, 2013). Indeed, mechanical stress applied to mammalian cells has been shown to spread through the actin cytoskeleton and onto lamin proteins (Ramdas and Shivashankar, 2015; Tajik et al., 2016). These forces change chromatin conformation by stretching it and lead to increased expression levels in affected chromosome regions. In plants, both abiotic and biotic stressors may change the mechanical force applied on a cell. For example, appressoria of fungal plant pathogens induce strong mechanical forces on cells (Bastmeyer et al., 2002). These may be transduced *via* the NE into the nucleus and lead to changes in chromosome organisation and gene expression.

Future research investigating the interplay between the NE and gene transcription in plants may expand its focus from *A. thaliana* to other species and move from whole-plant and organ-level to cell type and single cell level. *A. thaliana* is characterised by its relatively small and gene-dense genome. Plants with larger genomes and lower gene density are likely to exhibit different pattern of NE associated chromatin and gene expression. This would follow the significant differences in chromosome organisation identified in species, such as wheat, maize, and tomato compared to *A. thaliana* (Dong et al., 2017; Concia et al., 2020). Furthermore, elucidating the NE proteome and the NE associated chromatin on cell-type specific level and in response to biotic and abiotic challenges may lead to the identification of functionally important NE associated chromatin anchors and chromatin modifying complexes. These may become targets for manipulation of gene expression in plants. Future studies may further resolve which locus-specific features cause some genes to be upregulated upon transfer to the nuclear periphery and some genes to be downregulated. These may be accompanied by investigations aiming to shed light on the molecular mechanisms involved in the movement of loci towards and away from the periphery and how flanking genes are affected by gene-positioning at the NE. In human cell lines, for example, about 50–100 Kb of flanking chromatin is detached from the nuclear lamina upon activation of single genes (Brueckner et al., 2020). It remains to be seen whether plant genomes with diverse sizes and gene densities exhibit a similar pattern of chromatin detachment and how this affects the transcription of neighbouring genes.

## Author Contributions

All authors wrote the manuscript. HWN developed the concept and edited the manuscript. All authors contributed to the article and approved the submitted version.

### Conflict of Interest

The authors declare that the research was conducted in the absence of any commercial or financial relationships that could be construed as a potential conflict of interest.
